# Comparison of sample characteristics in two pregnancy cohorts: community-based versus population-based recruitment methods

**DOI:** 10.1186/1471-2288-13-149

**Published:** 2013-12-06

**Authors:** Brenda MY Leung, Sheila W McDonald, Bonnie J Kaplan, Gerald F Giesbrecht, Suzanne C Tough

**Affiliations:** 1 Department of Community Health Sciences, University of Calgary, Calgary, AB, Canada; 2 Child Development Centre, Alberta Children’s Hospital, Calgary, AB, Canada; 3 Department of Pediatrics, University of Calgary, Calgary, AB, Canada

**Keywords:** Recruitment strategy, Community-based, Population-based, Cohort studies, Participant characteristics

## Abstract

**Background:**

One of the biggest challenges for population health studies is the recruitment of participants. Questions that investigators have asked are “who volunteers for studies?” and “does recruitment method influence characteristics of the samples?” The *purpose* of this paper was to compare sample characteristics of two unrelated pregnancy cohort studies taking place in the same city, in the same time period, that employed different recruitment strategies, as well as to compare the characteristics of both cohorts to provincial and national statistics derived from the Maternity Experiences Survey (MES).

**Methods:**

One pregnancy cohort used community-based recruitment (e.g. posters, pamphlets, interviews with community media and face-to-face recruitment in maternity clinics); the second pregnancy cohort used both community-based and population-based (a centralized system identifying pregnant women undergoing routine laboratory testing) strategies.

**Results:**

The pregnancy cohorts differed in education, income, ethnicity, and foreign-born status (p < 0.01), but were similar for maternal age, BMI, and marital status. Compared to the MES, the lowest age, education, and income groups were under-represented, and the cohorts were more likely to be primiparous.

**Conclusions:**

The findings suggest that non-stratified strategies for recruitment of participants will not necessarily result in samples that reflect the general population, but can reflect the target population of interest. Attracting and retaining young, low resource women into urban studies about pregnancy may require alternate and innovative approaches.

## Background

Recruitment of participants is often one of the biggest challenges for population health studies, regardless of study purpose, design, or outcome. The relative success of multiple types of recruitment strategies has been previously assessed. Webster and colleagues evaluated the recruitment techniques used in a pregnancy study and found a combination of active (e.g., advertising) and passive (e.g., word of mouth) techniques to be effective [1]. However, they also stated that the resultant study sample was less ethnically diverse, more affluent, and more educated than the population of their catchment area, and thus additional methods would be required in the future to obtain a more representative sample [1]. Patterson and colleagues provided a framework for the design and implementation of successful recruitment activities that helped recruiters increase access to the target population and foster negotiating skills [2]. Sanders and colleagues emphasized the importance of comprehensive recruitment programs with multiple strategies employed simultaneously, combined with ongoing assessment of the success of each strategy [3].

For observational studies such as longitudinal cohorts, a major challenge is recruiting participants who are representative of the target population, so that study findings can be generalized to the population of interest. Golding and Birmingham described lessons learned from previous cohort studies such as the Danish National Birth Cohort study, the Norwegian MoBa study, the Generation R study, and The Avon Longitudinal Study of Parents and Children [4]. Some of the recommendations they listed were using personal contact at enrolment, ensuring recruitment personnel are appropriate and engaged in the study, and using positive enthusiastic promotional material [4]. Strategies worthy of investment from the outset include raising the profile of the study and ensuring clear, comprehensive, and confidential communication with potential participants [4].

With sampling and subject selection issues considered, questions that arise are “who volunteers for studies?” and “does recruitment method affect composition of the samples?” There are a number of well-established cohorts world-wide, from Great Britain to Australia, China to the U.S. However, few studies have reported on how much the cohort sample reflected the population from which the samples are drawn. One study that did so was the Generation R study, which compared their sample to the Rotterdam population using variables established by Statistics Netherlands [5]. The investigators found that overall the Generation R participants tended to have a higher socio-economic status (SES) than the general population [5]. Thus, the research results may not be generalizable to the population at large, and caution should be taken with interpreting outcomes.

Recently, two cohort studies recruited pregnant women within the same urban centre (Calgary, AB, a city of approximately one million people), and around the same period of time (2008 – 2012): the Alberta Pregnancy Outcomes and Nutrition (APrON) study (http://www.apronstudy.ca) and the All Our Babies (AOB) study. The two studies used different methods of recruitment, which enabled us to compare the strategies that were implemented within the same context. APrON’s method was entirely community-based: pregnant women were approached in maternity and radiology clinics, local businesses, community events, and through city-wide media coverage. The AOB study initially employed a community-based recruitment strategy, and then changed to a population-based strategy in collaboration with the city’s clinical laboratory services, which effectively gave the study access to all pregnant women in Calgary who presented to a physician for medical care. The AOB and APrON community-based strategies were not identical which permitted further comparison between types of community-based recruitment, in addition to comparing community-based to population-based recruitment strategies.

The *purpose* of this paper was to determine the extent to which different recruitment strategies in two unrelated pregnancy cohort studies taking place in the same city in the same time period affected the resulting sample characteristics. Comparisons were made between the samples, as well as to provincial and national statistics derived from the Maternity Experiences Survey (MES) [6] (see description of MES under “Methods”). The profile of the MES served as a standard, or ‘target population’ of women and families having children in Alberta and Canada. The specific objectives were to:

1. Describe the recruitment strategies in the APrON and AOB cohort studies;

2. Compare the sociodemographic characteristics of participants in the two studies;

3. Compare the sample characteristics of APrON and AOB to the MES provincial and national survey samples.

### About the cohort studies

*APrON study.* The APrON study is a prospective pregnancy cohort study whose primary focus is to investigate the role of the intrauterine nutrient environment. The primary questions addressed by the APrON study relate to prenatal maternal nutrition as a predictor of maternal mental health (depression and anxiety), birth outcomes (prematurity, congenital anomalies), and long-term child development (neurodevelopment, behaviour, cognitive health).

*AOB study.* The AOB study is also a prospective pregnancy cohort study, whose goal is to examine maternal well-being during the perinatal period and infant outcomes such as preterm birth, and to identify the current barriers and facilitators to accessing prenatal care in Calgary. The AOB study is following maternal-infant pairs across the early life course to further examine risk and protective factors important for child developmental outcomes and maternal well-being.

## Methods

### Recruitment strategy for APrON

APrON started recruiting in the spring of 2009, and continued until July 2012. Recruitment initially took place in the two major city centres of Alberta (Calgary and Edmonton), but by 2011 about 85% of the sample was from Calgary, so resources for subsequent recruitment were redirected to only Calgary. Recruitment methods differed a little between the two cities, but were consistently community-based. As AOB recruited only in Calgary, the APrON recruitment strategies discussed in this article are based on the activities that took place in Calgary only.

APrON formulated a comprehensive community-based plan to recruit participants [7]. First, high volume maternity clinics were identified and contacted. Research assistants (RAs) were physically present in the waiting areas of clinics that agreed to participate, and approached pregnant women about the study. Radiology clinics were then added as sites for recruitment, also using RAs. At some clinics, nurses recruited on behalf of APrON and were paid $10 per referral. A Public Relations Coordinator was hired to attend community events (e.g., wedding fairs, baby fairs, community festivals), and to negotiate with local businesses to have APrON posters and brochures on display. Multiple APrON investigators were interviewed and featured in newspapers and on television, which attracted attention to the website and project. Although recruitment began in a relatively high socioeconomic (SES) area of the city, attempts to diversify the demographic make-up of the sample resulted in expansion to quadrants of the city with higher proportions of lower-income and new Canadians. To make it easier for women from immigrant/refugee backgrounds to be included in the study, and not have to travel the distance to the primary APrON office, satellite clinics were set up for data collection (including collection of biological specimens) in several physicians’ offices. APrON also made use of social media by setting up Facebook and Twitter accounts.

### Recruitment strategy for AOB study

There were two recruitment phases in the AOB study, the first beginning in 2008 and the second in 2009. The objectives of the first phase (which is referred to as the Observational Cohort (OC)) were to examine health care utilization and maternal well-being across the perinatal period. For the second phase (which is referred to as the Prediction Cohort (PC)) the focus expanded to examine biological and environmental markers for spontaneous preterm delivery. Women were recruited to the OC from health care offices, using community posters, word-of-mouth, and through the regional health services website. Women were recruited to the PC using a collaborative strategy with the laboratory service. In Alberta, clinical practice guidelines for prenatal care stipulate viral serology testing for all pregnant women by public health laboratories. In Calgary, this service is provided by Calgary Laboratory Service (CLS), whose lists for prenatal viral serology tests are continually updated as newly pregnant women enter into prenatal care. All women who received prenatal viral serology testing in Calgary during study recruitment (2009–2011) were initially contacted by CLS, who asked permission to release patient contact information to the AOB research staff [8]. Women who consented were telephoned by an AOB research assistant to determine their eligibility.

Table 1 lists the recruitment activities undertaken by APrON and AOB, and examples of places and events where the studies were publicized. Table 2 provides the inclusion criteria for each cohort.

**Table 1:** Community-based recruitment strategies in the APrON and AOB studies

**What**	**Where**	**APrON examples**	**AOB examples**
In-person	Maternity and radiology (ultrasound) clinics community events	Research assistants (RAs) stationed in waiting rooms of high volume medical clinics or doctors’ offices; Nurses recruited on behalf of APrON;	Onsite and telephone recruitment by RAs at low-risk maternity care practices, and research nurse onsite at an obstetrician maternity practice
		RAs attend local community events such as festivals, baby fairs, wedding fairs; RAs gave presentations at prenatal and nutrition classes; RAs were present at the babies’ products section of a major department store.	
Posters, pamphlets	Public places, businesses, community places	Posters at yoga studios, health food stores, clothing stores (especially those for pregnant women and children); posters and brochures in drug stores, bookstores, childcare facilities, coffee shops, fitness centers, retail stores, grocery stores, libraries, beauty/hair salons, work sites, places of worship, and family practice and pediatrician offices	Posters at family practice and pediatrician offices
Print media	Newspapers	Stories published in local newspapers, magazine	
Advertisement	Television, radio	PI interviewed by journalists; video produced by the communications department at the University	
Social media	Internet	Website (http://www.apronstudy.ca), Facebook page, Twitter account; website link put on websites of community supporters	
Media interviews with investigators	Articles	Published in Swerve magazine, Calgary Child, Insite, AHS newsletter, Calgary Herald, Sun, Metro, Apple and the Birthing Magazine.	
	Taped interviews	Appearances on Global, CBC and various radio stations	
Satellite/mobile clinics	Doctors office	Offices that saw high volume of women from diverse ethnic background	
	High school for pregnant teens		
Other		Word of mouth	Word of mouth

**Table 2:** Comparison of APrON and AOB selection criteria

**Selection criteria**	**APrON**	**AOB-OC**	**AOB-PC**
Maternal age	≥16 years	≥18 years	≥18 years
Language	Able to complete questionnaires in English	Able to complete questionnaires in English	Able to complete questionnaires in English
Gestational age at enrollment	<27 weeks	<24 wks	<17wks
Specific criteria	Not planning to move out of the city within 6 months of inclusion into study	Receiving prenatal care	Nulliparous or primiparous OR personal or familial history of preterm birth;
			Receiving prenatal care;
			Singleton pregnancy

### Provincial and National Statistics from the Maternity Experiences Survey

The *Maternity Experiences Survey* (MES) was the first of its kind to examine the pregnancy, labour, birth and postpartum experiences of Canadian women. The survey was sponsored by the Canadian Perinatal Surveillance System of the Public Health Agency of Canada (PHAC) (see http://www.phac-aspc.gc.ca/rhs-ssg/pdf/survey-eng.pdf) [6].

The MES was a cross sectional sample survey, using post-census data from the 2006 Canadian Census of Population to identify babies born between the target dates, as well as the mothers of those babies. The MES sample was stratified by province or territory, and the mother’s age. The sample frame was further refined in some provinces by mother’s residence in a census metropolitan area, or the presence of other children in the household. Mothers aged less than 20 years at the time of their babies’ birth were oversampled. A simple random sample was selected without replacement within each stratum. The final sample consisted of 8,542 women from across Canada, inclusive of all provinces and territories, 6,421 of whom had complete questionnaire information for analyses. Data was collected during the period of October 23, 2006 to January 31, 2007 [6].

### Data analysis

Cross tabulations were performed using Chi^2^ tests to compare the characteristics of the APrON cohort with AOB-OC, APrON with AOB-PC, and AOB-OC with AOB-PC. Due to multiple comparisons, we set the criteria for statistical significance at p < 0.01 for the Calgary cohort comparisons.

## Results

The number of APrON participants was 2200 when recruitment ended in July 2012; the number of AOB participants was 3300 when recruitment ceased in 2011. As not all data are entered at the time of this writing, the sample sizes available for analysis in this paper were APrON = 1200, AOB-OC = 1118, and AOB-PC = 1878. These samples were non-overlapping; i.e., women in the AOB-PC sample were not represented in the AOB-OC sample. APrON and AOB-OC share some similarity in recruitment strategies, namely in-person contact and use of posters. However, APrON’s community based strategy was more extensive, utilizing multiple sources and media types, while AOB-OC focused mainly on perinatal care clinics (Table 1). The inclusion criteria for both APrON and AOB were similar (Table 2). Table 3 summarizes the maternal sociodemographic characteristics of the APrON and AOB cohorts compared to the MES provincial and national pregnancy samples.

**Table 3:** Sociodemographic characteristics of the APrON & AOB cohorts compared to the MES provincial and national samples

**Characteristic**	**APrON**	**AOB-OC**	**AOB-PC**	**MES Alberta (n=651)**	**MES Canada (n=6,421)**	**Calgary cohort comparisons**	p-value**
Age	n=1,200	n=916	n=1,455			Overall Chi^2^	0.196
15–19	0.4 (0.05-0.78)	0.5 (0.07-1.02)	0.4 (0.08-0.74)	3.8 (3.3–4.3)	3.0 (2.8–3.2)	AOB-OC vs. APrON	0.198
20–24	4.5 (3.3-5.7)	5.4 (4.0-6.9)	6.2 (4.9-7.4)	15.2 (12.8–17.6)	13.0 (12.3–13.8)	AOB-PC vs. APrON	0.507
25–29	27.1 (24.6-29.6)	30.5 (27.5-33.4)	26.5 (24.2-28.7)	34.1 (31.6–36.6)	33.1 (32.2–33.9)	AOB-OC vs. AOB-PC	0.095
30–34	44.6 (41.8-47.4)	41.7 (38.5-44.9)	42.9 (40.3-45.4)	30.6 (28.2–32.9)	32.9 (32.0–33.8)		
35–39	19.9 (17.6-22.2)	19.7 (17.1-22.2	20.1 (18.1-22.2)	13.0 (10.8–15.3)	14.5 (13.7–15.3)		
≥40	3.5 (2.5-4.5)	2.2 (1.2-3.1)	3.9 (2.9-4.9)	2.6 (1.4–3.7)	3.0 (2.5–3.4)		
Education	n=1,154	n=1,117	n=1,875			Overall Chi^2^	<0.001
Less than high school	1.5 (0.8-2.2)	3.3 (2.3-4.4)	3.1 (2.3-3.9)	7.3 (5.6–9.1)	7.6 (6.9–8.2)	AOB-OC vs. APrON	<0.001
High school graduate	8.2 (6.6-9.8)	18.1 (15.8-20.3)	22.8 (20.9-24.7)	22.8 (19.7–25.9)	19.2 (18.2–20.1)	AOB-PC vs. APrON	<0.001
Postsecondary education	90.3 (88.6-92.0)	78.6 (76.2-81.0)	74.1 (72.1-76.1)	69.5***	72.1***	AOB-OC vs. AOB-PC	0.009
Income	n=1,147	n=1,083	n=1,820			Overall Chi^2^	0.008
At or below the LICO✝	5.9 (4.6-7.3)	9.2 (7.5-11.0)	8.6 (7.3-9.9)	13.4 (10.9-15.8)	18.4 (17.4–19.4)	AOB-OC vs. APrON	0.003
Above the LICO✝✝	94.1 (92.7-95.4)	90.8 (89.0-92.5)	91.4 (90.1-92.7)	77.8 (74.7-80.8)	72.6 (71.5–73.7)	AOB-PC vs. APrON	0.008
						AOB-OC vs. AOB-PC	0.543
Parity	n=1,177	n=1,112	n=1,866			Overall Chi^2^	<0.001
Primiparous	56.8 (53.9-59.6)	54.9 (51.9-57.8)	46.5 (44.2-48.7)	46.0 (42.4–49.7)	44.7 (44.0–45.5)	AOB-OC vs. APrON	0.361
Multiparous	43.2 (40.4-46.1)	45.1 (42.2-48.1)	53.5 (51.3-55.8)	53.8 (50.2–57.4)	54.9 (54.1–55.6)	AOB-PC vs. APrON	<0.001
						AOB-OC vs. AOB-PC	<0.001
BMI (mean (95% CI))	n=1171	n=1,094	n=1,838			Overall F stat	<0.001
Pre-pregnancy	24.9 (24.6 - 25.2)	23.7 (23.4-23.9)	24.7 (24.5-25.0)	24.4 (24.0–24.8)	24.4 (24.3–24.6)	AOB-OC vs. APrON	0.005
						AOB-PC vs. APrON	0.011
						AOB-OC vs. AOB-PC	<0.001
						Overall F stat	<0.001
Postpartum	n = 935	n=1008	n=1632			AOB-OC vs. APrON	0.001
	25.6 (25.3 - 25.9)	24.9 (24.7-25.2)	26.0 (25.7-26.3)	25.5 (25.1–25.9)	25.4 (25.2–25.5)	AOB-PC vs. APrON	0.106
						AOB-OC vs. AOB-PC	<0.001
Marital status	n=1,158	n=1,115	n=1,876			Overall Chi^2^	0.010
Married/common law	96.3 (95.2-97.4)	93.5 (92.1-95.0)	95.2 (94.2-96.2)	Not available	Not available	AOB-OC vs. APrON	0.003
Single/divorced/separated	3.7 (2.6-4.8)	6.5 (5.0-7.9)	4.8 (3.8-5.8)			AOB-PC vs. APrON	0.156
						AOB-OC vs. AOB-PC	0.052
Born in Canada	n=1,152	n=1,118	n=1,878			Overall Chi^2^	0.007
Yes	81.3 (79.1-83.6)	76.1 (73.6-78.6)	79.7 (77.8-81.5)	Not available	Not available	AOB-OC vs. APrON	0.002
No	18.7 (16.4-20.9)	23.9 (21.4-26.4)	20.3 (18.5-22.2)			AOB-PC vs. APrON	0.260
						AOB-OC vs. AOB-PC	0.023
Ethnicity	n=1,143	n=1,114	n=1,876			Overall Chi^2^	<0.001
Caucasian	86.4 (84.4-88.3)	75.1 (72.6-77.7)	81.3 (79.6-83.1)	Not available	Not available	AOB-OC vs. APrON	<0.001
Non-Caucasian	13.6 (11.7-15.7)	24.9 (22.3-27.4)	18.7 (16.9-20.4)			AOB-PC vs. APrON	<0.001
						AOB-OC vs. AOB-PC	<0.001

Note: Except for BMI, all values are percentages and 95% CI.

**p-values derived from Chi^2^ tests for categorical variables and ANOVA/independent t-tests (F stat) for BMI. The overall Chi^2^ p-value compared the three cohorts within each characteristic variable; we set the p-value = 0.01 for statistical significance to account for multiple comparisons.

***Education categories did not match across the cohorts, including MES. Education categories were therefore collapsed to facilitate comparisons; thus some MES stats for collapsed categories do not contain confidence intervals.

✝Low income cut-off (LICO) MES category which corresponds to APrON and AOB category of < $40,000.

✝✝Low income cut-off (LICO) MES category which corresponds to APrON and AOB category of ≥ $40,000.

### Calgary cohort comparisons

The test results were mixed for the various group comparisons (see Table 3). The omnibus Chi 2 p-values and F test p-values for categorical and continuous variables, respectively, were ≤ 0.01 for all comparisons with the exception of maternal age. Pairwise comparisons showed that the women in APrON and AOB-OC were similar for age and parity, but differed for education levels, income, BMI, marital status, being born in Canada, and ethnicity (p < 0.01). Compared to women in AOB-OC, women in APrON were more likely to be married, have higher education and income levels, be Canadian born and Caucasian, and have higher pre/post pregnancy BMI.

Compared to AOB-PC, APrON participants were more likely to have higher education and income levels, be primiparous, and significantly less likely to be born in Canada and non-Caucasian (p < 0.01). The two cohorts were similar in age, marital status and BMI profile.

Women in AOB-OC were more likely to have higher education levels, be non-Caucasian, and have higher pre-pregnancy and postpartum BMIs than women in AOB-PC (p < 0.01).

### Target population comparisons

In general, the proportions for the various characteristics of the cohorts were not similar to the data from the MES (see Table 3), indicating that the recruitment strategies used by the pregnancy cohorts did not replicate the stratified approach used by the MES. Compared to the MES, the lowest age, education, and income groups were under-represented, and the cohorts were more likely to be primiparous. Nevertheless, comparisons with other data sources at the local and provincial level such as administrative data on perinatal health and census community profiles during or close to the study time period suggest that the APrON and AOB participants are generally representative of the pregnancy and parenting population at the local (city) and provincial levels. For example, the average age of women in Calgary and Alberta giving birth in 2010 was 30.8 and 29.5 years, respectively [9]. In the Calgary cohorts, the average age at delivery was approximately 30 years. Approximately one-quarter of women in Calgary were foreign-born and one-quarter were a visible minority according to the Canadian Census [10], with only slightly lower percentages seen in the Calgary cohorts (Table 3). Furthermore, approximately 50% of women in the APrON and AOB studies reported a household income of over 100 K, which aligns with the median income of couple families according to recent statistics from Statistics Canada for 2010 (approximately 97 K) [11].

## Discussion

This study compared the characteristics of women who were recruited for pregnancy cohorts in the same city during the same time period but using different recruitment strategies. Comparisons were also made to a national study that used a stratified sampling frame and to provincial and local birth statistics during the study time period. The differences in the characteristics of women recruited in the Calgary cohorts were associated with recruitment approach. We cannot rule out the possibility that the content of the research projects held different appeal for different women and may have contributed to sample differences. In general, these findings suggest that even with different recruitment methods, women who participate in longitudinal projects on pregnancy tend to reflect the middle part of the bell curve of the population from which they are drawn, in that they are close to the mean for maternal age, and reflect median levels of education and income. Indeed, the proportions of educated and affluent women were high in all three Calgary cohorts. The AOB-OC cohort had the highest proportion of non-Caucasians, which is a direct reflection of the neighborhood in which community-based recruitment began, specifically the ethnically diverse northeast quadrant of the city which comprises immigrants and people belonging to many cultural groups.

Although the Calgary cohorts were more similar than different in demographic profile, they did not capture the degree of variability seen in the MES sample, particularly characteristics of women having children in rural communities or smaller urban centres, which, as noted above, likely reflects the non-stratified sampling approach of the Calgary cohorts compared to the Census sampling strategy employed by the MES. The MES assured its external validity by oversampling women less than 20 years of age. Comparison with the MES suggests that generalizability of results to some strata of childbearing women (e.g., women under 20) may be limited in the Calgary cohorts. Nevertheless, the Calgary cohorts achieved representativeness of the community/population to which they intend to generalize results, namely pregnant women and families with young children living in Canadian urban centres.

It is worth noting that within the characteristics of both the APrON and AOB cohorts, variability of predictor and outcome variables is not synonymous with variability in sociodemographic background [12]. Despite some overrepresentation of higher SES, there was wide variability within the predictors and outcomes, which did not affect each study’s ability to address its research (outcome) questions [13]: in the case of APrON, the relationship between nutrition and maternal mental health, or in the case of AOB, factors related to prenatal care or adverse birth outcomes. This suggests that the research questions under investigation do not have socioeconomic boundaries, akin to the robust associations found in the well-known Adverse Childhood Experiences (ACE) study among a relatively affluent sample of over 17,000 Americans [14]. Results from the ACE study showed that in a middle-class sample, adverse childhood experiences are common and are a prime determinant of long-term health status [14].

Comparisons between independently constructed cohorts have inherent limitations. First, the community based recruitment activities for APrON and AOB were disproportionate (i.e. APrON had more activities than AOB); therefore, some of the differences between the two Alberta cohorts are to be expected. Second, the selection criteria differed for APrON and AOB which may have emphasized different characteristics within the respective cohorts. Third, because our analyses are by definition *post hoc*, we faced the challenge of comparing variables that had been collected in slightly different ways. Despite these limitations, the sampling methods used to recruit our cohorts are sufficient for providing insight into risk associations and are likely the most feasible approach for many pregnancy cohort studies [15]. Nevertheless, future cohort studies that adopt a non-stratified sampling approach would benefit from strategies that enhance diversity in participant characteristics to ensure external validity.

The recruitment strategies of the APrON and AOB studies reflect the importance of commitment in the planning phases for high response rates and low attrition rates. While preliminary comparisons between continuers and discontinuers in the AOB study suggest that attrition was related to lower socioeconomic status and poorer mental health, which is consistent with other cohort studies [4,16,17], no difference was found in the APrON study. The recruitment strategies used in both APrON and AOB were diverse and comprehensive, which were congruent with methods discussed in the literature (e.g. word of mouth, promotional materials, personal contact, recruitment sites and locations of target population) [1,3], in addition to using a city-wide public health laboratory service provider. Further strategies were used for cohort retention and ongoing engagement including newsletters and incentives for participation (APrON and AOB), as well as social media links and annual meetings for participants known as “baby parties” (APrON). Both cohorts ensured that research staff members were well-trained and maintained high research standards. These steps build confidence and trust between study participants and study staff. The effectiveness of different types of recruitment strategies (e.g., word of mouth, local media, clinic-based) in the APrON study has been published elsewhere [7].

## Conclusion

In summary, the results of these analyses underscore the importance of sampling approach in study design for both internal and external validity considerations. Our results show that community and city-wide (i.e., laboratory) approaches can result in sample sizes that are large enough to allow for subanalysis of population characteristics (e.g., parity, ethnicity), and samples that are similar to the urban landscape where the studies were established. However, researchers would do well to specifically target vulnerable groups who tend to be under-represented in research in general. Thus approaches for future studies attempting to correct the under-representation of vulnerable groups might consider focusing on recruitment and retention of women who are marginalized in terms of age, language, income, and education. Specialized strategies will be required to meet the needs of these women.

## Competing interests

The authors declare that they have no competing interests.

## Authors’ contributions

BL and SM wrote the initial draft of the manuscript, and revised various drafts to produce the completed draft. SM performed the statistical tests for comparing the characteristics of the cohorts. BK and GG collaborated in developing the framework and analyses for the manuscript. BK, GG, and ST reviewed and edited the manuscript. All co-authors participated in manuscript preparation. All authors read and approved the final manuscript.

## Pre-publication history

The pre-publication history for this paper can be accessed here:

http://www.biomedcentral.com/1471-2288/13/149/prepub
